# Evaluation of metabolic dysfunction-associated fatty liver disease using FibroScan, diet, and microbiota: A large cross-sectional study

**DOI:** 10.1371/journal.pone.0277930

**Published:** 2022-11-23

**Authors:** Tetsuyuki Tateda, Chikara Iino, Takafumi Sasada, Satoshi Sato, Go Igarashi, Shogo Kawaguchi, Kenichiro Mikami, Tetsu Endo, Kaori Sawada, Tatsuya Mikami, Shinsaku Fukuda, Shigeyuki Nakaji, Hirotake Sakuraba

**Affiliations:** 1 Department of Gastroenterology, Hirosaki University Graduate School of Medicine, Hirosaki, Japan; 2 Department of Internal Medicine, Owani Hospital, Owani, Japan; 3 Department of Gastroenterology and Internal Medicine, Mutsu General Hospital, Mutsu, Japan; 4 Center of Healthy Aging Innovation, Hirosaki University Graduate School of Medicine, Hirosaki, Japan; Auburn University, UNITED STATES

## Abstract

**Objective:**

We evaluated the clinical characteristics of metabolic dysfunction-associated fatty liver disease (MAFLD) to evaluate the usefulness of the MAFLD diagnostic criteria in a resident health survey.

**Methods:**

In 1056 participants of a health survey, we compared obesity, diabetes, metabolic dysregulation, FibroScan-aspartate aminotransferase (FAST) score, dietary habits, and gut microbiota between healthy individuals and participants with MAFLD and Nonalcoholic fatty liver disease (NAFLD).

**Results:**

The proportion of participants with MAFLD in the fatty liver was higher than that with NAFLD (88.1% vs. 75.5%, respectively). Of 36 participants with a FAST score > 0.35, 29 (80.6%) participants had MAFLD and 23 (63.9%) participants had NAFLD. Of 29 patients with liver fibrosis, 26 (89.7%) participants had obesity and metabolic dysregulation. In the evaluation of diet, the total energy, protein, dietary fiber, and salt intake were significantly higher in participants with MAFLD than those in participants without fatty liver. In the microbiota analysis, the results of the linear discriminant analysis effect size analysis revealed nine bacterial genera that were significantly different in participants with MAFLD in comparison with participants without fatty liver. Of these genera, the relative abundance of *Blautia* was especially low in participants with MAFLD.

**Conclusion:**

In a resident health survey, participants with MAFLD had a higher proportion of fatty liver than those with NAFLD. MAFLD criteria could help in improved screening of participants with liver fibrosis. Therefore, the MAFLD criteria could be a useful diagnostic tool for aggressively identifying participants with a high risk of fatty liver. Additionally, *Blautia* might be involved in the development of MAFLD.

## Introduction

Nonalcoholic fatty liver disease (NAFLD)/nonalcoholic steatohepatitis (NASH) is one of the most common chronic liver diseases. The incidence of NAFLD is increasing globally; its reported prevalence is approximately 20%–46% in Europe and the United States [1, 2] and 9%–30% in Japan [3]. In recent years, it has been reported that metabolic disorders, such as obesity and diabetes are associated with various events in patients with NAFLD [4–7]. However, the NAFLD diagnostic criteria do not include metabolic disorders. In 2020, a new diagnostic criterion for fatty liver—metabolic dysfunction-associated fatty liver disease (MAFLD)—was proposed [8]. The advantages of MAFLD diagnostic criteria are that metabolic disorders are included in them, they are independent of the amount of alcohol consumption, and they can be combined with other liver diseases. MAFLD is an appropriate term for defining fatty liver disease associated with metabolic dysregulation. The modification of “NAFLD” to “MAFLD” would help understand the role of metabolic dysfunction in the disease. Although the usefulness of MAFLD has been demonstrated, the prevalence and clinical characteristics of patients with MAFLD remain unclear. Furthermore, there is insufficient evidence to demonstrate the usefulness of the diagnostic criteria.

In this study, we compared obesity, diabetes, metabolic dysregulation, FibroScan-aspartate aminotransferase (FAST) score [9], dietary habits, and gut microbiota between healthy individuals and participants with MAFLD and NAFLD to clarify the characteristics of MAFLD.

## Materials and methods

### Study participants

A total of 1056 adults (age range 20–88 years) who participated in the Iwaki Health Promotion Project held in June 2018 in Aomori Prefecture, northern Japan, were included. This study was performed in accordance with the ethical standards of the Declaration of Helsinki and was approved by the Hirosaki University Medical Ethics Committee (Authorization number: 2018–063). Written informed consent was obtained from all participants.

### Transient elastography

Transient elastography with liver stiffness measurement (LSM) and controlled attenuation parameter (CAP) measurements were performed using a FibroScan 530 compact device equipped with both M and XL probes. The examinations were performed by five well-trained hepatology specialists. When the number of measurements was less than 10 or the ratio of the interquartile range was greater than 0.30, the measured values were excluded due to unreliability. Fatty liver was diagnosed if CAP ≥ 248 dB/m [10]. The FAST score was calculated using the following formula [9]: ex/(1 + ex), where x = −1.65+1.07×ln (LSM)+2.66×10−8×CAP3−63.3×AST−1. Based on previous reports, liver fibrosis was defined as a FAST score > 0.35 [9].

### Clinical parameters

The following clinical parameters were recorded on the morning of transient elastography: sex, age, height, weight, waist circumference, body mass index (BMI; calculated by dividing the weight in kilograms by the squared height in meters), hepatitis B surface (HBs) antigen or anti-hepatitis C virus (HCV) antibody, and serum levels of total protein, albumin, total bilirubin (T-bil), AST, alanine aminotransferase (ALT), gamma-glutamyl transpeptidase (GGT), blood glucose, insulin, hemoglobin A1c (HbA1c), uric acid, total cholesterol, high-density lipoprotein (HDL)-cholesterol, low-density lipoprotein (LDL)-cholesterol, triglyceride, platelets, and C-reactive protein (CRP). Type 2 diabetes was defined as either fasting blood glucose ≥ 126 mg/dL and HbA1c ≥ 6.5% or the use of oral hypoglycemic drugs. Insulin resistance index was calculated using the homeostasis model assessment of insulin resistance (HOMA-IR) as follows: fasting blood glucose (mg/dL) × fasting insulin (μU/mL)/405 [11]. The FIB-4 index [12], NFS [13], and fatty liver index (FLI) [14] were calculated using the following formulas according to previous reports.

FIB-4 index = {age (years)×AST (U/L)}/{platelets (×109/L)×√ALT}. NFS = −1.675+0.037×age (years)+0.094×BMI (kg/m2)+1.13×impaired glucose tolerance/diabetes (yes = 1, no = 0)+0.99×AST/ALT−0.013×platelets (×109/L)−0.66×albumin (g/dL)

FLI = ey/(1 + ey)×100, y = 0.953×ln {triglyceride (mg/dL)}+0.139×{BMI (kg/m2)}+0.718×ln {γ-GTP (U/L)}+0.053×{waist circumference (cm)}–15.745

**Fig 1:**
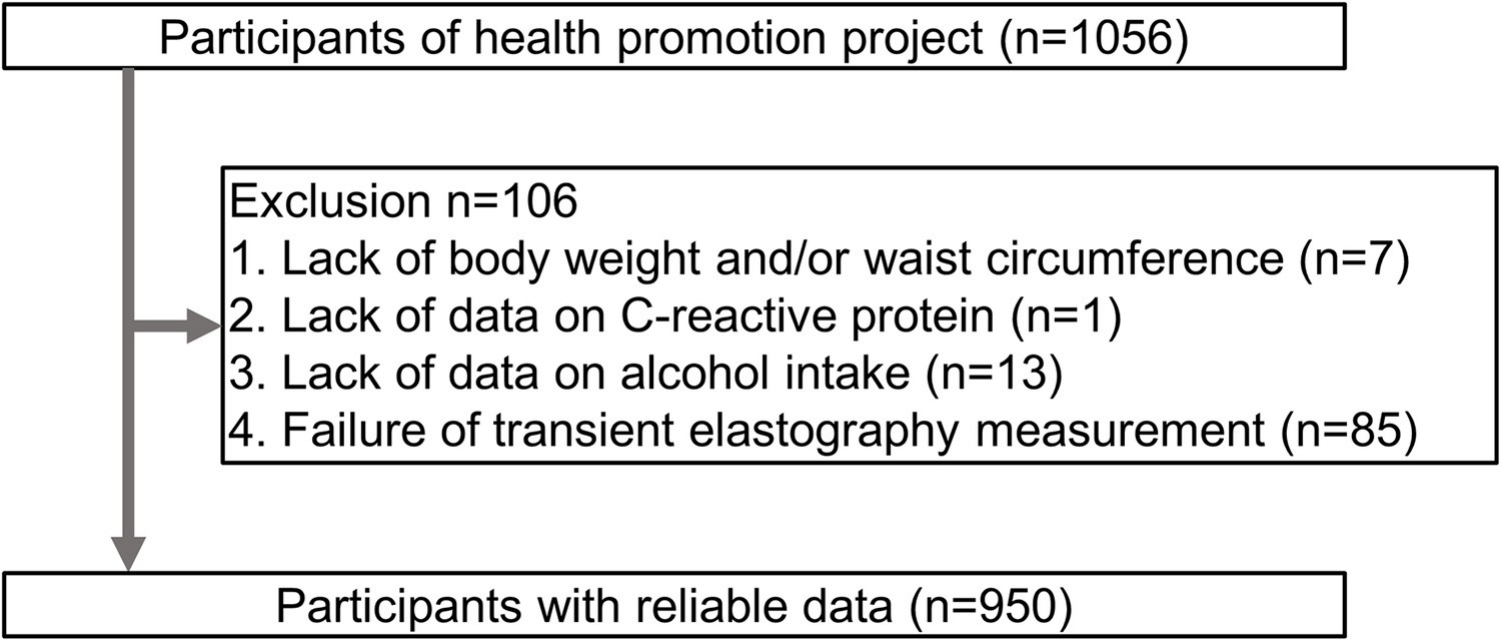
Flowchart for participant selection.

Energy and nutrient intakes were calculated based on the results of a brief self-administered diet history questionnaire (BDHQ) [15]. The BDHQ is a convenient diet assessment questionnaire developed in Japan that asks the frequency and amount of consumption of beer, wine, whisky, Japanese sake and Japanese distilled spirits and includes questions concerning the intake frequency of 58 foods and beverages commonly consumed in Japan [15]. We excluded participants with lacking data regarding body weight and/or waist circumference (n = 7), serum CRP level (n = 1), alcohol intake (n = 13), and transient elastography (n = 85) (**Fig 1**).

### Classification of fatty liver

Based on a previous report, in participants with fatty liver based on CAP measurements, those meeting any of the following criteria were diagnosed with MAFLD [16]: obesity (BMI ≥ 23 kg/m2); type 2 diabetes; or BMI < 23 kg/m2 with ≥ two metabolic dysregulations: waist circumference ≥ 90 cm in men and ≥ 80 cm in women, blood pressure ≥ 130/85 mmHg or specific drug treatments, triglyceride ≥ 150 mg/dL or specific drug treatment, HDL-cholesterol < 40 mg/dL in men and < 50 mg/dL in women or specific drug treatment, impaired glucose tolerance (fasting blood glucose ≥ 100 mg/dL or HbA1c ≥ 5.7%), homeostasis model assessment of insulin resistance (HOMA-IR) ≥ 2.5, and CRP ≥ 2 mg/dL. NAFLD was defined as fatty liver on transient elastography in the absence of the following: excessive alcohol consumption (≥ 30 g/day in men and ≥20 g/day in women), HBs antigen or HCV antibody positivity, and use of steatogenic medications such as amiodarone, methotrexate, corticosteroids, and tamoxifen. The participants who satisfied both the MAFLD and NAFLD criteria were defined to have an overlap, and participants who did not meet either of the criteria were defined as non-MAFLD/non-NAFLD. The participants were divided into five groups: no fatty liver (normal), MAFLD, NAFLD, overlap, and non-MAFLD/non-NAFLD. To evaluate the effects of risk factors on liver fibrosis, the participants with MAFLD were divided into three groups according to the number of risk factors corresponding to the MAFLD diagnostic criteria.

### DNA extraction from fecal samples

Fecal samples were collected from each subject in specific containers (TechnoSuruga Laboratory Co., Ltd., Shizuoka, Japan). The samples were suspended in guanidine thiocyanate solution [100 mM Tris-HCl (pH 9.0), 40 mM Tris–EDTA (pH 8.0), and 4 M guanidine thiocyanate] and stored at -80°C until DNA extraction. Frozen fecal solids were beaten with zirconia beads at 5 m/s for 2 min using FastPrep 24 (MP Biomedicals, Santana Ana, CA, USA). DNA was extracted from 200 μL of the suspension using a Magtration System 12 GC (Precision System Science, Japan) and MagdDEA DNA 200 (Precision System Science) as the reagent for automatic nucleic acid extraction.

### Next-generation sequence analysis and 16S rDNA-based taxonomic analysis

Fecal samples were analyzed for a series of representative bacteria in the human gut microbiota using primers for the V3-V4 hypervariable region of the 16S rDNA of prokaryotes, according to previous studies [17, 18]. Sequencing was performed using an Illumina MiSeq system (Illumina, San Diego, CA, USA). Quality filtering was performed as follows: only reads that had quality value scores ≥ 20 for more than 99% of the sequence were extracted for the analysis. Bacterial sequences were detected and identified using Metagenome@KIN software (World Fusion Co., Tokyo, Japan) and the TechnoSuruga Lab Microbial Identification database DB-BA 10.0 (TechnoSuruga Laboratory) at 97% sequence similarity. A linear discriminant analysis effect size (LEfSe) was performed to compare the microbiota features between the MAFLD and non-fatty liver groups (Normal). LEfSe scores measure the consistency of differences in relative abundance between taxa in both groups with a higher score indicating higher consistency.

### Statistical analysis

Statistical analyses of clinical data were performed using EZR [19]. The Mann–Whitney U test was used for comparisons between two groups of continuous variables, and one-way analysis of variance was used for comparisons between multiple groups. Fisher’s exact test was used to compare ratios between groups. Spearman’s rank correlation coefficients were used to correlate two continuous variables, and logistic regression analysis was used for multivariate analysis of binary variables. A p-value<0.05 was considered statistically significant. LEfSe combines the Kruskal–Wallis test or pairwise Wilcoxon rank-sum test with linear discriminant analysis (LDA). LEfSe also ranks the features by effect size, thus identifying features that explain most of the biological differences. LEfSe analysis was performed using the following conditions: α = 0.01, and threshold of the logarithmic LDA score for discriminative features = 2.0 [20].

## Results

### Characteristics of participants

**Fig 2:**
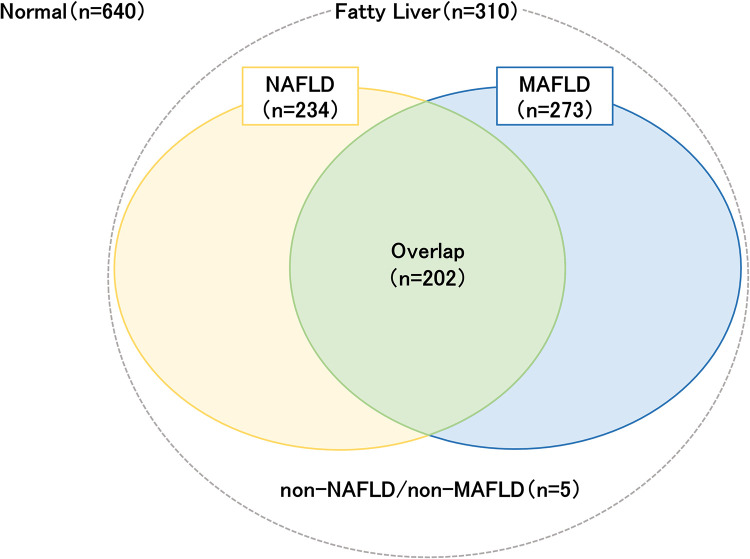
Grouping of the study participants.

Of the 950 participants, 402 (42.3%) participants were male. The median age of the cohort was 52 (39–65) years, and the median BMI, waist circumference, alcohol intake, and HbA1c were 22.5 (20.2–24.8) kg/m2, 83.8 (76.9–90.3) cm, 1.17 (0–17.57) g/day, and 5.7% (5.4–5.9%), respectively (**Table 1**). Based on the CAP values, 310 subjects were diagnosed with fatty liver. The percentage of participants with hypertension and diabetes was 44% and 5.9%, respectively. In non-invasive scores, the median values were as follows: fatty liver index: 14.4 (6.0–36.4); CAP, 219 (185–262) dB /m; LSM, 4.3 (3.5–5.4) kPa, and FAST score, 0.058 (0.030–0.103). Healthy, MAFLD, NAFLD, overlap, and non-MAFLD/non-NAFLD groups included 640, 273, 234, 202, and 5 participants, respectively (Fig **2**).

**Table 1:** Characteristics of participants.

	All	Normal	MAFLD	NAFLD	Overlap	Fatty liver with non MAFLD and non NAFLD
n	950	640	273	234	202	5
Age (years)	52 (39–65)	51 (38–64)	58 (44–68)	58 (42–68)	59 (44–68)	56 (49–60)
Gender, Male (%)	402 (42.3%)	244 (38.13%)	144 (52.7%)	97 (41.5%)	86 (42.6%)	3 (60%)
BMI (kg/m^2^)	22.5 (20.2–24.8)	21.5 (19.6–23.4)	25.2 (23.5–27.5)	24.6 (22.5–27.0)	25.2 (23.4–27.6)	22.2 (21.5–22.4)
Waist circumference (cm)	83.8 (76.9–90.3)	80.4 (74.9–86.4)	91.1 (86.5–97.5)	89.4 (85–96)	91.0 (86.0–97.1)	81.4 (80.5–83.4)
Diabetes (%)	56 (5.9%)	19 (2.97%)	37 (13.6%)	29 (12.4%)	29 (14.36%)	0 (0%)
Hypertension (%)	418 (44%)	239 (37.3%)	174 (63.7%)	121 (51.7%)	118 (58.4%)	2 (40%)
Alcohol intake (g/day)	1.17 (0–17.57)	1.2 (0–17.6)	1.3 (0–20.8)	0 (0–4.8)	0 (0–4.2)	38.5 (33.1–50.8)
Platelet count (×10^4^/μL)	25.7 (22.5–29.8)	25.9 (22.4–29.5)	25.3 (22.4–30.0)	25.4 (22.8–30.7)	25.3 (22.8–30.7)	23.2 (22.9–25.0)
BUN (mg/dL)	13.6 (11.2–16.3)	13.7 (11.2–16.2)	13.5 (11.4–16.8)	13.3 (11.3–16.6)	13.5 (11.5–17.0)	12.9 (10.6–15.7)
Uric acid (mg/dL)	4.9 (4.1–6.0)	4.7 (4.0–5.7)	5.5 (4.7–6.5)	5.0 (4.2–6.1)	5.1 (4.4–6.1)	5.5 (4.9–5.5)
Creatinine (mg/dL)	0.64 (0.55–0.76)	0.63 (0.54–0.75)	0.67 (0.57–0.77)	0.65 (0.56–0.76)	0.65 (0.58–0.76)	0.69 (0.64–0.81)
Total bilirubin (mg/dL)	0.8 (0.7–1.0)	0.8 (0.7–1.0)	0.8 (0.6–1)	0.8 (0.6–1.0)	0.8 (0.6–1.0)	1.1 (0.8–1.5)
AST (U/L)	21 (17–25)	20 (17–24)	23 (19–29)	21 (18–27)	22 (19–28)	19 (19–20)
ALT (U/L)	18 (13–25)	16 (12–22)	23 (17–36)	22 (16–33)	23 (16.3–36)	15 (12–16)
GGT (U/L)	21 (15–38)	20 (14–32)	30 (19–53)	24.5 (18–38.8)	26 (19–39.8)	22 (20–28)
Total protein (g/dL)	7.1 (6.8–7.3)	7.1 (6.8–7.3)	7.1 (6.9–7.4)	7.1 (6.9–7.4)	7.1 (6.9–7.4)	6.9 (6.6–6.9)
Albumin (g/dL)	4.4 (4.2–4.6)	4.4 (4.2–4.6)	4.4 (4.2–4.6)	4.4 (4.2–4.6)	4.4 (4.2–4.6)	4.3 (4.3–4.4)
HbA1c (%)	5.7 (5.4–5.9)	5.6 (5.4–5.8)	5.8 (5.6–6.1)	5.8 (5.6–6.1)	5.9 (5.6–6.2)	5.5 (5.3–5.6)
HOMA-IR	1.10 (0.81–1.57)	0.98 (0.72–1.29)	1.59 (1.12–2.37)	1.57 (1.11–2.38)	1.67 (1.22–2.6)	0.72 (0.71–0.90)
Glucose (mg/dL)	92 (86–99.8)	90 (84–96)	97 (91–108)	95 (89–107.8)	97 (91–110.8)	93 (92–96)
Total cholesterol (mg/dL)	202 (178–225)	199 (175–224)	207 (88–228)	204 (184.3–227.8)	186.3 (186.3–228)	207 (194–209)
Triglyceride (mg/dL)	79 (56–114)	69 (51–99)	105 (79–154)	94.5 (67–130)	97 (73–141)	55 (54–56)
LDL-cholesterol (mg/dL)	116 (95–136)	113 (92–134)	121 (106–141)	120 (106–141.8)	121 (108–142)	109 (103–133)
HDL-cholesterol (mg/dL)	63 (54–75)	66 (56–79)	57 (48–66)	58 (48–70)	56.5 (47–67.8)	71 (70–88)
M2BPGi C.O.I	0.51 (0.36–0.72)	0.48 (0.32–0.67)	0.61 (0.44–0.82)	0.61 (0.43–0.80)	0.65 (0.46–0.83)	0.4 (0.4–0.65)
NAFLD fibrosis score	-2.13 (-3.22 - -1.09)	-2.27 (-3.39 - -1.27)	-1.64 (-2.77 - -0.58)	-1.93 (-2.82 - -0.71)	-1.59 (-2.77 - -0.57)	-1.95 (-2.09 - -1.00)
FIB-4 index	0.94 (0.66–1.42)	0.93 (0.65–1.41)	1.00 (0.68–1.47)	0.95 (0.63–1.43)	1.01 (0.65–1.48)	1.21 (1.21–1.23)
Fatty liver index	14.4 (6.0–36.4)	9.4 (4.3–21.3)	42.3 (21.0–64.3)	30.4 (15.3–55.2)	35.0 (18.8–59.1)	9.3 (8.4–10.1)
CAP (dB/m)	219 (185–262.8)	197 (166–220)	288 (266–313)	285 (263–309)	288 (264.3–312.8)	271 (262–272)
LSM (kPa)	4.3 (3.5–5.4)	4.1 (3.4–5.2)	4.6 (3.8–5.8)	4.6 (3.7–5.8)	4.6 (3.7–5.9)	3.8 (3.6–3.9)
FAST score	0.058 (0.030–0.103)	0.046 (0.024–0.081)	0.100 (0.057–0.205)	0.084 (0.045–0.181)	0.091 (0.050–0.189)	0.052 (0.048–0.056)
FAST score>0.35	36 (3.8%)	6 (0.9%)	29 (10.6%)	23 (9.8%)	22 (10.9%)	0 (0%)

Data are presented as number (percentage) or median (range). ALT, alanine aminotransferase; AST, aspartate aminotransferase; BMI, body mass index; BUN, blood urea nitrogen; CAP, controlled attenuation parameter; FAST, FibroScan-aspartate aminotransferase; FIB-4, fibrosis-4; GGT, γ-glutamyl transpeptidase; HbA1c, hemoglobin A1c; HDL, high density lipoprotein; HOMA-IR, homeostasis model assessment of insulin resistance; LDL, low density lipoprotein; LSM, liver stiffness measurement; NAFLD, nonalcoholic fatty liver disease; MAFLD, metabolic dysfunction-associated fatty liver disease; M2BPGi C.O.I, macrophage galactose-specific lectin-2 binding protein glycosylation isomer cut off index

### Examination of MAFLD

**Fig 3:**
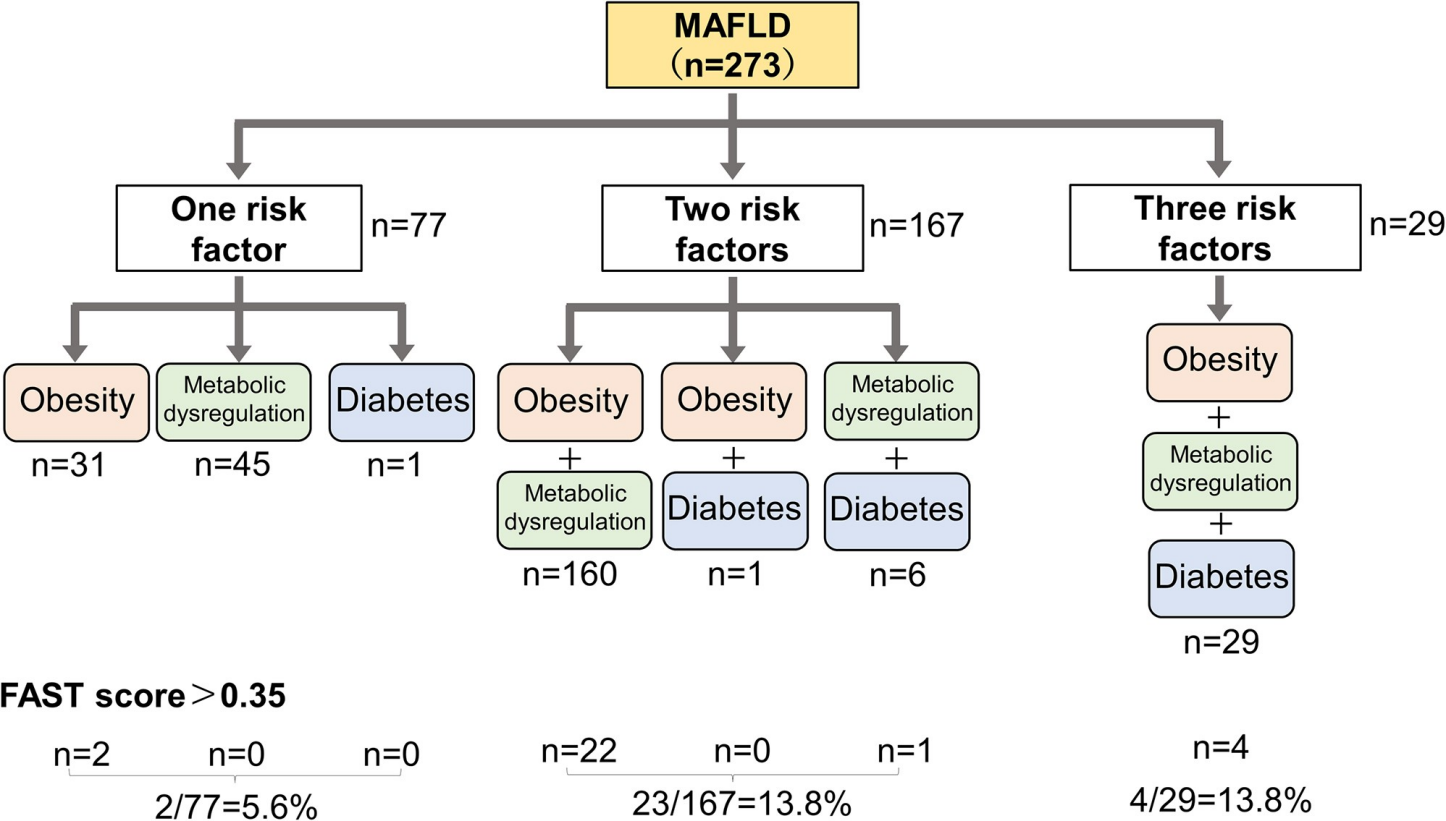
Flowchart of risk factors and number of risk factors in MAFLD.

**Fig 4:**
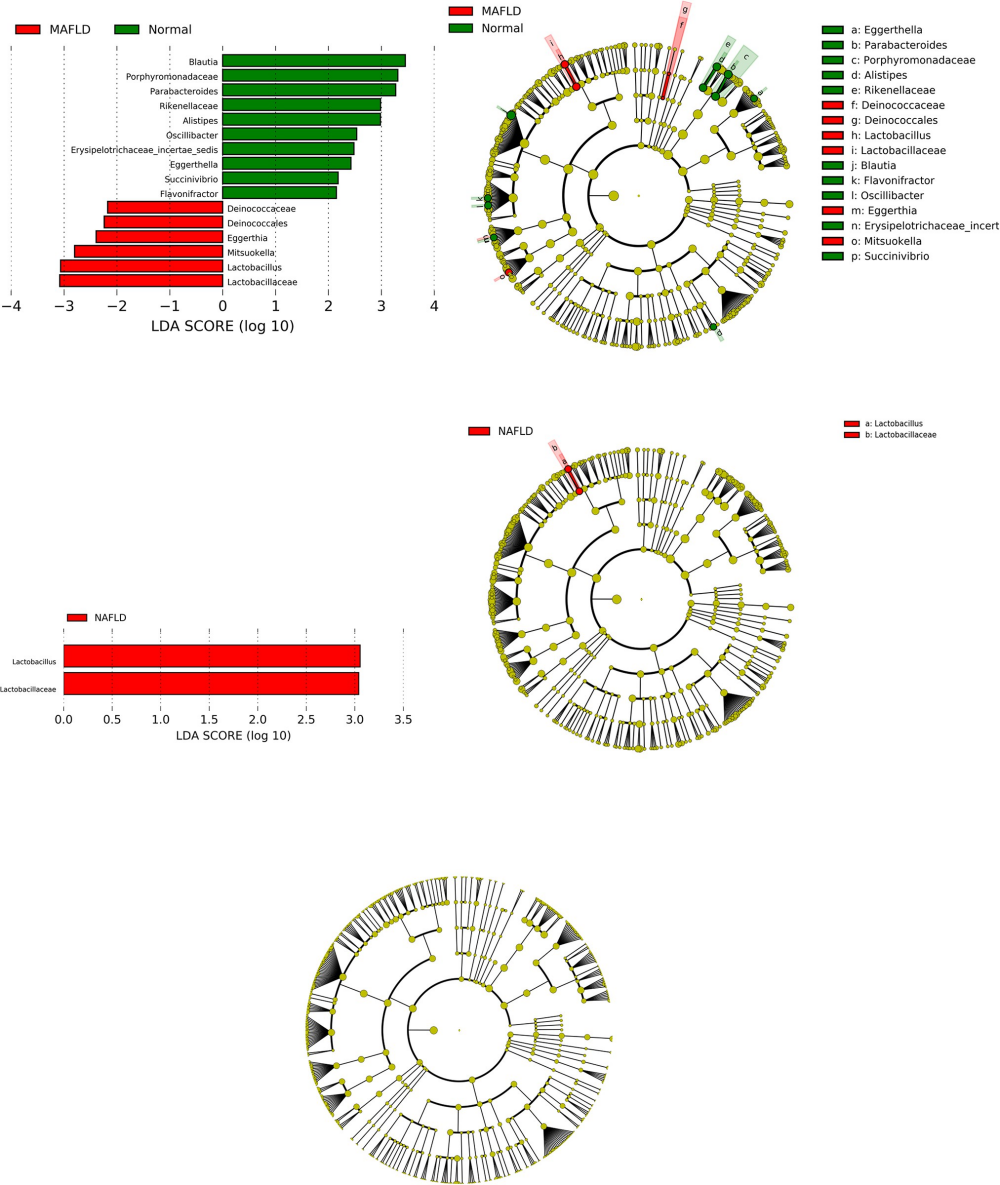
a: Result of the LEfSe analysis between MAFLD and healthy participants. b: Result of LEfSe analysis of participants with and without NAFLD. c: Result of LEfSe analysis of participants with NAFLD and MAFLD.

In comparison with participants without fatty liver (healthy), those with MAFLD had significant differences in age, percentage of males, hepatobiliary enzyme, indices of liver fibrosis and steatosis, and items related to metabolic abnormalities, which are included in MAFLD criteria (**Table 2**). BMI, waist circumference, prevalence of hypertension and diabetes, fatty liver index, and serum levels of uric acid, γ-GTP, and triglycerides were significantly higher in participants with MAFLD than those in participants with NAFLD. Furthermore, the proportion of participants with MAFLD in the fatty liver was higher than that with NAFLD (88.1% vs. 75.5%, respectively). When participants with MAFLD were divided into three groups according to the number of risk factors of MAFLD diagnostic criteria, participants with one, two, and three risk factors included 77 (28.2%), 167 (61.2%), and 29 (10.6%) participants, respectively. In factor-specific examination, obesity, diabetes, and metabolic dysregulation were noted in 221 (81.0%), 37 (13.6%), and 240 (87.1%) participants, respectively. A total of 106 (38.8%) participants had both obesity and metabolic dysregulation, and the combination was the most frequent (**Fig 3**). In comparison with the number of risk factors, the indices of liver steatosis—such as the fatty liver index and CAP—increased as the number of factors increased (**Table 3**). To examine the effects of diet on MAFLD, we found that the total intake of energy, protein, dietary fiber, and salt in participants with MAFLD was significantly higher than that in participants without fatty liver (**Table 4**). Additionally, we evaluated and compared the energy and nutrient intake between normal subjects and subjects with NAFLD (**Table 5**) and between subjects with MAFLD and NAFLD (**Table 6**) using the BDHQ. There were no significant differences in energy and nutrient intake in all groups; however, alcohol intake was significantly different between participants with MAFLD and NAFLD. The results of LEfSe analysis revealed six bacterial genera that were overrepresented and three genera that were underrepresented in participants with MAFLD compared with those without fatty liver (**Fig 4A**). Of these genera, the relative abundance of *Blautia* was especially low in participants with MAFLD. Meanwhile, *Lactobacillus* and *Lactobacillaceae* were more abundant in participants with MAFLD than that in those with NAFLD (**Fig 4B**). After excluding 202 overlapping participants, LEfSe analysis did not reveal any significant differences between participants with MAFLD and NAFLD (**Fig 4C**).

**Table 2:** Comparisons of MAFLD, healthy, and NAFLD groups.

	MAFLD	Normal	NAFLD	p Value	*p* Value
				MAFLD vs. Normal	MAFLD vs. NAFLD
n	273	640	234		
Age (years)	58 (44–68)	51 (38–64)	58 (42–68)	<0.01	0.471
Gender, Male (%)	144 (52.7%)	244 (38.13%)	97 (41.5%)	<0.01	<0.05
BMI (kg/m^2^)	25.2 (23.5–27.5)	21.5 (19.6–23.4)	24.6 (22.5–27.0)	<0.01	<0.05
Waist circumference (cm)	91.1 (86.5–97.5)	80.4 (74.9–86.4)	89.4 (85–96)	<0.01	<0.05
Diabetes (%)	37 (13.6%)	19 (2.97%)	29 (12.4%)	<0.01	0.791
Hypertension (%)	174 (63.7%)	239 (37.3%)	121 (51.7%)	<0.01	<0.05
Alcohol intake (g/day)	1.34 (0–20.77)	1.2 (0–17.6)	0 (0–4.8)	0.845	<0.05
Platelet count (×10^4^/μL)	25.3 (22.4–30.0)	25.9 (22.4–29.5)	25.4 (22.8–30.7)	0.935	0.422
BUN (mg/dL)	13.5 (11.4–16.8)	13.7 (11.2–16.2)	13.3 (11.3–16.6)	0.625	0.691
Uric acid (mg/dL)	5.5 (4.7–6.5)	4.7 (4.0–5.7)	5.0 (4.2–6.1)	<0.01	<0.05
Creatinine (mg/dL)	0.67 (0.57–0.77)	0.63 (0.54–0.75)	0.65 (0.56–0.76)	<0.01	0.176
Total bilirubin (mg/dL)	0.8 (0.6–1)	0.8 (0.7–1.0)	0.8 (0.6–1.0)	<0.05	0.57
AST (U/L)	23 (19–29)	20 (17–24)	21 (18–27)	<0.01	0.079
ALT (U/L)	23 (17–36)	16 (12–22)	22 (16–33)	<0.01	0.078
GGT (U/L)	30 (19–53)	20 (14–32)	24.5 (18–38.8)	<0.01	<0.05
Total protein (g/dL)	7.1 (6.9–7.4)	7.1 (6.8–7.3)	7.1 (6.9–7.4)	0.314	0.95
Albumin (g/dL)	4.4 (4.2–4.6)	4.4 (4.2–4.6)	4.4 (4.2–4.6)	0.528	0.886
HbA1c (%)	5.8 (5.6–6.1)	5.6 (5.4–5.8)	5.8 (5.6–6.1)	<0.01	0.844
HOMA-IR	1.59 (1.12–2.37)	0.98 (0.72–1.29)	1.57 (1.11–2.38)	<0.01	0.909
Glucose (mg/dL)	97 (91–108)	90 (84–96)	95 (89–107.8)	<0.01	0.169
Total cholesterol (mg/dL)	207 (88–228)	199 (175–224)	204 (184.3–227.8)	<0.01	0.518
Triglyceride (mg/dL)	105 (79–154)	69 (51–99)	94.5 (67–130)	<0.01	<0.05
LDL-cholesterol (mg/dL)	121 (106–141)	113 (92–134)	120 (106–141.8)	<0.01	0.892
HDL-cholesterol (mg/dL)	57 (48–66)	66 (56–79)	58 (48–70)	<0.01	0.38
M2BPGi C.O.I	0.61 (0.44–0.82)	0.48 (0.32–0.67)	0.61 (0.43–0.80)	<0.01	0.529
Fatty liver index	42.30 (21.03–64.29)	9.43 (4.34–21.29)	30.38 (15.31–55.24)	<0.01	<0.05
FIB-4 index	1.00 (0.68–1.47)	0.93 (0.65–1.41)	0.95 (0.63–1.43)	0.22	0.327
CAP (dB/m)	288 (266–313)	197 (166–220)	285 (263–309)	<0.01	0.406
LSM (kPa)	4.6 (3.8–5.8)	4.1 (3.4–5.2)	4.6 (3.7–5.8)	<0.01	0.507
FAST score	0.100 (0.057–0.205)	0.046 (0.024–0.081)	0.084 (0.045–0.181)	<0.01	0.073
FAST score>0.35	29 (10.6%)	6 (0.9%)	23 (9.8%)	<0.01	0.883

Data are presented as number (percentage) or median (range). ALT, alanine aminotransferase; AST, aspartate aminotransferase; BMI, body mass index; BUN, blood urea nitrogen; CAP, controlled attenuation parameter; FAST, FibroScan-aspartate aminotransferase; FIB-4, fibrosis-4; GGT, γ-glutamyl transpeptidase; HbA1c, hemoglobin A1c; HDL, high density lipoprotein; HOMA-IR, homeostasis model assessment of insulin resistance; LDL, low density lipoprotein; LSM, liver stiffness measurement; M2BPGi C.O.I, macrophage galactose-specific lectin-2 binding protein glycosylation isomer cut off index

**Table 3:** Comparison by number of risk factors in MAFLD.

Number of risk factor in MAFLD	One	Two	Three	*p* Value
n	77	167	29	
Age (years)	56 (44–66)	59 (44–68)	65 (44–69)	0.447
Gender, Male (%)	32 (41.6%)	97 (58.1%)	15 (51.7%)	0.055
BMI (kg/m^2^)	22.7 (21.8–24.1)	25.9 (25.9–28.1)	26.6 (25.1–30.2)	<0.01
Waist circumference (cm)	85.2 (82.7–88.6)	94.2 (89.2–100.0)	94.4 (90.9–98.8)	<0.01
Diabetes (%)	1 (1.3%)	7 (4.2%)	29 (100%)	<0.01
Hypertension (%)	35 (45.5%)	116 (69.5%)	23 (79.3%)	<0.01
Alcohol intake (g/day)	1.14 (0–20.58)	2.23 (0–21.28)	0 (0–14.64)	0.976
Platelet count (×10^4^/μL)	25.4 (23.4–29.9)	25.3 (22.3–30.0)	23.2 (20.3–31.2)	0.387
BUN (mg/dL)	13.6 (11.3–16.6)	13.2 (11.4–16.75)	14.3 (12.4–18.3)	0.148
Uric acid (mg/dL)	5.0 (4.2–5.9)	5.9 (4.8–6.9)	5.2 (4.4–5.9)	<0.01
Creatinine (mg/dL)	0.67 (0.57–0.75)	0.69 (0.58–0.78)	0.64 (0.59–0.79)	<0.05
Total bilirubin (mg/dL)	0.9 (0.7–1.0)	0.8 (0.6–1.0)	0.8 (0.6–1.0)	0.768
AST (U/L)	21 (19–26)	24 (20–30.5)	20 (18–24)	0.059
ALT (U/L)	21 (14–25)	27 (19–40)	23 (17–35)	<0.01
γ-GTP (U/L)	21 (16–43)	31 (21–55)	32 (21–53)	0.141
Total protein (g/dL)	7.1 (6.8–7.3)	7.1 (6.9–7.4)	7.0 (6.9–7.4)	0.964
Albumin (g/dL)	4.5 (4.3–4.6)	4.4 (4.2–4.6)	4.2 (4.0–4.4)	<0.01
HbA1c (%)	5.6 (5.4–5.8)	5.8 (5.6–6.1)	7.5 (6.6–8.8)	<0.01
HOMA-IR	1.21 (0.99–1.51)	1.75 (1.25–2.40)	2.99 (1.81–4.55)	<0.01
Glucose (mg/dL)	93 (87–99)	97 (92–106)	139 (122–155)	<0.01
Total cholesterol (mg/dL)	208 (190–239)	207 (185.5–225)	204 (186–223)	0.13
Triglyceride (mg/dL)	97 (71–140)	109 (81–156)	124 (89–184)	0.16
LDL-cholesterol (mg/dL)	121 (101–142)	121 (107–141)	121 (108–135)	0.871
HDL-cholesterol (mg/dL)	65 (55–75)	55 (47–63.5)	52 (40–56)	<0.01
M2BPGi C.O.I	0.59 (0.42–0.74)	0.59 (0.44–0.82)	0.78 (0.67–0.95)	<0.01
NAFLD fibrosis score	-2.22 (-2.96 - -1.34)	-1.48 (-2.63 - -0.51)	-0.46 (-2.02 - -0.19)	<0.01
FIB-4 index	1.00 (0.69–1.44)	0.97 (0.69–1.48)	1.22 (0.65–1.57)	0.274
Fatty liver index	21.0 (13.1–32.3)	50.0 (31.6–69.1)	54.6 (41.7–86.1)	<0.01
CAP (dB/m)	271 (260–287)	294 (269.5–318)	317 (281–339)	<0.01
LSM (kPa)	4.1 (3.3–4.9)	4.8 (4.1–6.1)	5.6 (4.6–7.4)	<0.01
FAST score	0.075 (0.044–0.116)	0.132 (0.065–0.225)	0.1137 (0.061–0.224)	<0.01
FAST≧0.35	2 (2.6%)	23 (13.8%)	4 (13.8%)	<0.05

Data are presented as number (percentage) or median (range). ALT, alanine aminotransferase; AST, aspartate aminotransferase; BMI, body mass index; BUN, blood urea nitrogen; CAP, controlled attenuation parameter; FAST, FibroScan-aspartate aminotransferase; FIB-4, fibrosis-4; GGT, γ-glutamyl transpeptidase; HbA1c, hemoglobin A1c; HDL, high density lipoprotein; HOMA-IR, homeostasis model assessment of insulin resistance; LDL, low density lipoprotein; LSM, liver stiffness measurement; M2BPGi C.O.I, macrophage galactose-specific lectin-2 binding protein glycosylation isomer cut off index

**Table 4:** Comparison of energy and nutrient intake between normal subjects and subjects with MAFLD.

	Normal	MAFLD	*p* Value
n	640	273	
Energy intake (kcal/kg・IBW)	30.8 (25.6–37.4)	32.3 (26.4–39.5)	<0.05
Carbohydrate (g/kg・IBW)	4.0 (3.2–5.0)	4.1 (3.5–5.1)	0.067
Total fat (g/kg・IBW)	0.89 (0.70–1.11)	0.88 (0.69–1.17)	0.779
Animal-based fat (g/kg・IBW)	0.40 (0.31–0.54)	0.42 (0.30–0.57)	0.326
Plant-based fat (g/kg・IBW)	0.47 (0.36–0.61)	0.47 (0.34–0.59)	0.508
Total protein (g/kg・IBW)	1.11 (0.93–1.47)	1.24 (0.94–1.52)	<0.05
Animal-based protein (g/kg・IBW)	0.62 (0.48–0.85)	0.67 (0.47–0.93)	0.123
Plant-based protein (g/kg・IBW)	0.49 (0.39–0.61)	0.53 (0.42–0.64)	<0.05
Saturated fatty acid (g/kg・IBW)	0.23 (0.17–0.30)	0.23 (0.17–0.31)	0.82
Monounsaturated fatty acid (g/kg・IBW)	0.31 (0.25–0.40)	0.31 (0.24–0.41)	0.993
Polyunsaturated fatty acid (g/kg・IBW)	0.22 (0.18–0.28)	0.23 (0.18–0.29)	0.544
Cholesterol (mg/kg・IBW)	6.1 (4.4–8.2)	6.5 (4.3–8.6)	0.562
Total dietary fiber (g/kg・IBW)	0.17 (0.13–0.22)	0.19 (0.14–0.25)	<0.05
Soluble dietary fiber (g/kg・IBW)	0.043 (0.032–0.059)	0.047 (0.034–0.062)	0.05
Insoluble dietary fiber (g/kg・IBW)	0.12 (0.10–0.16)	0.13 (0.10–0.17)	<0.05
Salt (g/kg・IBW)	0.18 (0.14–0.22)	0.19 (0.15–0.24)	<0.05
Ethanol (g/kg・IBW)	0.021 (0–0.28)	0.021 (0–0.36)	0.896

Data are presented as median (range). IBW, ideal body weight; MAFLD, metabolic dysfunction-associated fatty liver disease

**Table 5:** Comparison of energy and nutrient intake between normal subjects and subjects with MAFLD.

	Normal	NAFLD	*p* Value
n	640	234	
Energy intake (kcal/kg・IBW)	30.8 (25.6–37.4)	31.6 (25.4–38.4)	0.908
Carbohydrate (g/kg・IBW)	4.0 (3.2–5.0)	4.1 (3.5–5.1)	0.141
Total fat (g/kg・IBW)	0.89 (0.70–1.11)	0.90 (0.72–1.17)	0.373
Animal-based fat (g/kg・IBW)	0.40 (0.31–0.54)	0.43 (0.31–0.57)	0.191
Plant-based fat (g/kg・IBW)	0.47 (0.36–0.61)	0.47 (0.36–0.60)	0.968
Total protein (g/kg・IBW)	1.11 (0.93–1.47)	1.21 (0.93–1.54)	0.089
Animal-based protein (g/kg・IBW)	0.62 (0.48–0.85)	0.67 (0.47–0.95)	0.187
Plant-based protein (g/kg・IBW)	0.49 (0.39–0.61)	0.52 (0.41–0.63)	0.095
Saturated fatty acid (g/kg・IBW)	0.23 (0.17–0.30)	0.24 (0.18–0.31)	0.271
Monounsaturated fatty acid (g/kg・IBW)	0.31 (0.25–0.40)	0.32 (0.25–0.40)	0.546
Polyunsaturated fatty acid (g/kg・IBW)	0.22 (0.18–0.28)	0.23 (0.18–0.28)	0.490
Cholesterol (mg/kg・IBW)	6.1 (4.4–8.2)	6.5 (4.4–8.7)	0.303
Total dietary fiber (g/kg・IBW)	0.17 (0.13–0.22)	0.19 (0.14–0.25)	0.022
Soluble dietary fiber (g/kg・IBW)	0.043 (0.032–0.059)	0.049 (0.033–0.062)	<0.05
Insoluble dietary fiber (g/kg・IBW)	0.12 (0.10–0.16)	0.14 (0.10–0.17)	<0.05
Salt (g/kg・IBW)	0.18 (0.14–0.22)	0.18 (0.15–0.24)	0.057
Ethanol (g/kg・IBW)	0.02 (0–0.28)	0.00 (0–0.08)	<0.05

Data are presented as median (range). IBW, ideal body weight; NAFLD, nonalcoholic fatty liver disease

**Table 6:** Comparison of energy and nutrient intake between subjects with MAFLD and NAFLD.

	MAFLD	NAFLD	*p* Value
n	273	234	
Energy intake (kcal/kg・IBW)	32.3 (26.4–39.5)	31.6 (25.4–38.4)	0.11
Carbohydrate (g/kg・IBW)	4.1 (3.5–5.1)	4.1 (3.5–5.1)	0.842
Total fat (g/kg・IBW)	0.88 (0.69–1.17)	0.90 (0.72–1.17)	0.645
Animal-based fat (g/kg・IBW)	0.42 (0.30–0.57)	0.43 (0.31–0.57)	0.789
Plant-based fat (g/kg・IBW)	0.47 (0.34–0.59)	0.47 (0.36–0.60)	0.541
Total protein (g/kg・IBW)	1.24 (0.94–1.52)	1.21 (0.93–1.54)	0.908
Animal-based protein (g/kg・IBW)	0.67 (0.47–0.93)	0.67 (0.47–0.95)	0.996
Plant-based protein (g/kg・IBW)	0.53 (0.42–0.64)	0.52 (0.41–0.63)	0.82
Saturated fatty acid (g/kg・IBW)	0.23 (0.17–0.31)	0.24 (0.18–0.31)	0.471
Monounsaturated fatty acid (g/kg・IBW)	0.31 (0.24–0.41)	0.32 (0.25–0.40)	0.639
Polyunsaturated fatty acid (g/kg・IBW)	0.23 (0.18–0.29)	0.23 (0.18–0.28)	0.944
Cholesterol (mg/kg・IBW)	6.5 (4.3–8.6)	6.5 (4.4–8.7)	0.699
Total dietary fiber (g/kg・IBW)	0.19 (0.14–0.25)	0.19 (0.14–0.25)	0.865
Soluble dietary fiber (g/kg・IBW)	0.047 (0.034–0.062)	0.049 (0.033–0.062)	0.92
Insoluble dietary fiber (g/kg・IBW)	0.13 (0.10–0.17)	0.14 (0.10–0.17)	0.817
Salt (g/kg・IBW)	0.19 (0.15–0.24)	0.18 (0.15–0.24)	0.407
Ethanol (g/kg・IBW)	0.02 (0–0.36)	0.00 (0–0.08)	<0.05

Data are presented as median (range). IBW, ideal body weight; MAFLD, metabolic dysfunction-associated fatty liver disease; NAFLD, nonalcoholic fatty liver disease

### Evaluation of liver fibrosis using FAST score

A total of 36 participants had a FAST score > 0.35; of them, 29 (80.6%) participants had MAFLD, and 23 (63.9%) participants had NAFLD (**Table 1**). In the comparison of MAFLD according to the number of risk factors, 2 (5.6%) participants with one risk factor, 23 (13.8%) participants with two risk factors, and 4 (13.8%) participants with three risk factors had liver fibrosis (**Fig 3**). Of the 29 participants with liver fibrosis, 26 (89.7%) had obesity and metabolic dysregulation.

### Assessment of risk factors for liver fibrosis

Analysis of the correlation between the eight items associated with the diagnostic criteria for MAFLD and the FAST score revealed that only HDL-cholesterol was significantly negatively correlated, while the other seven items were significantly positively correlated (**Table 7**). In multivariate analysis, obesity (odds ratio, [OR]: 7.24, 95% confidence interval [CI]: 2.43–21.6, p<0.01) and metabolic dysregulation (OR: 3.19, 95% CI: 1.06–9.59, p = 0.039) were identified as independent factors associated with a FAST score > 0.35 (**Table 8**).

**Table 7:** Correlation with FAST score.

	r	*p* Value
BMI (kg/m^2^)	0.342	<0.01
HbA1c (%)	0.231	<0.01
Waist circumference (cm)	0.374	<0.01
Systolic blood pressure (mmHg)	0.359	<0.01
Triglyceride (mg/dL)	0.304	<0.01
HDL-cholesterol (mg/dL)	-0.094	<0.01
HOMA-IR	0.226	<0.01
CRP (mg/dL)	0.274	<0.01

BMI, body mass index; CRP, C-reactive protein; HbA1c, hemoglobin A1c; HDL, high density lipoprotein; HOMA-IR, homeostasis model assessment of insulin resistance

**Table 8:** Multivariate analysis results for risk factors with FAST score > 0.35.

Variables	Multivariable		
	OR	95% CI	*p* Value
Obesity	7.24	2.43–21.6	<0.01
Metabolic dysregulation	3.19	1.06–9.59	0.039
Diabetes	1.51	0.55–4.14	0.426

CI, confidence interval; OR, odds ratio

## Discussion

We found that, in participants with fatty liver, those with MAFLD had a higher proportion than those with NAFLD, and the criteria of MAFLD could identify participants with liver fibrosis more accurately than those with NAFLD in the resident health survey. Furthermore, multivariate analysis revealed that obesity and metabolic dysfunction are significant risk factors for liver fibrosis. In the evaluation of diet, total energy, protein, dietary fiber, and salt intake were significantly higher in participants with MAFLD than in those without fatty liver. Additionally, in microbiota analysis, a significant decrease in the relative abundance of *Blautia* was observed in those with MAFLD.

Our study found that the proportion of participants with MAFLD in the fatty liver group was higher than that of participants with NAFLD. A previous study in a resident health survey demonstrated that the prevalence of MAFLD in fatty liver was higher than that of NAFLD (94.3% vs. 73.8%, respectively) [21]. Furthermore, the study demonstrated that MAFLD criteria were superior to NAFLD criteria in detecting participants with significant liver fibrosis (68 vs. 48 of 2254 participants) [21]. Similarly, in the this study, the MAFLD criteria were able to detect more participants with liver fibrosis. The NAFLD criteria are based on the exclusion of alcohol-related diseases, autoimmune hepatitis, and viral hepatitis, whereas the MAFLD criteria are based on the inclusion of obesity, metabolic dysregulation, and diabetes, which are associated with liver fibrosis. Significant correlations were observed between the FAST score for predicting liver fibrosis and all items in the MAFLD criteria in the current study. Therefore, these results revealed the reason MAFLD criteria helped identify more participants at risk for liver fibrosis than the NAFLD criteria.

Obesity and metabolic dysregulation were significant independent factors for liver fibrosis in multivariate analysis in this study. A previous study found that obesity and weight gain increased the risk of liver fibrosis and that markers of liver fibrosis decreased in patients with weight loss [4]. Another study used transient elastography during health checkups and revealed that the complications of metabolic syndrome affected an increase in liver stiffness [5]. Furthermore, the current study revealed that approximately 90% of participants with MAFLD and liver fibrosis had obesity and metabolic dysregulation. Obesity and metabolic dysregulation are both closely associated with liver fibrosis. Therefore, in terms of detecting liver fibrosis, the MAFLD criteria could be superior to the NAFLD criteria in resident health surveys.

Diabetes was not a significant factor in the multivariate analysis of liver fibrosis in this study. However, recent evidence suggests that patients with diabetes are at a higher risk of developing liver fibrosis [22]. Previous studies have demonstrated that HbA1c was significantly higher in those with liver fibrosis than those without liver fibrosis [23], and HbA1c increased with the progression of liver fibrosis [24]. Furthermore, another study reported that insulin resistance plays an important role in the progression of liver fibrosis [25]. In the current study, diabetes was also a significant factor for liver fibrosis in univariate analysis and was correlated with the FAST score. However, diabetes was no longer a significant factor in the multivariate analysis after adjusting for obesity and metabolic dysregulation. This is because the participants in this health survey had better glycemic control and lower insulin resistance than those in other studies. Therefore, there were fewer participants with advanced liver fibrosis caused by diabetes in the healthy general population. According to a resident health survey, diabetes might not be a good indicator of liver fibrosis.

A study of the US population revealed that the prevalence of MAFLD and NAFLD is high (39.1% and 37.1%, respectively) [26]. Another study showed that the prevalence of liver steatosis was 33.7% [27], which is higher than that in our study. The difference may be attributed to the differences in the characteristics of subjects, such as BMI, ethnicity, laboratory parameters, and diagnostic cutoff value of CAP.

In this study, participants with MAFLD had a significantly higher total energy intake than those without fatty liver. A previous study that compared participants with NAFLD and those without fatty liver reported that those with NAFLD had a higher total energy intake [28]. This is closely associated with an increase in BMI, glucose intolerance, and metabolic dysregulation, which are the diagnostic criteria for MAFLD [29, 30]. Furthermore, salt intake was significantly higher in participants with MAFLD in the present study. Previous reports also found that excessive salt intake is associated with increased BMI, blood pressure, and waist circumference and increases the risk of NAFLD [31–33], BMI, blood pressure, and waist circumference were included in the MAFLD diagnostic criteria, and higher total energy and salt intake were associated with an increased risk of MAFLD.

LEfSe analysis demonstrated that the relative abundance of *Blautia* was significantly lower in participants with MAFLD in comparison with those without fatty liver, whereas its relative abundance was not significantly different between participants with MAFLD and NAFLD. Previous studies have demonstrated that *Blautia* produces acetic acid and butyric acid, which leads to a reduction in obesity by regulating G-protein-coupled receptors [34, 35]. Another study demonstrated that *Blautia* was inversely associated with the visceral fat area [36]. In the current study, the relative abundance of *Blautia* was lower and BMI was higher in participants with MAFLD in comparison with those without fatty liver. The relative abundance of *Blautia* could be associated with fat accumulation and BMI, which might be related to the pathogenesis of MAFLD.

This study had several limitations. First, we did not use histological evaluation with liver biopsy, which is the gold standard method for diagnosing fatty liver and liver fibrosis. However, a liver biopsy is an invasive procedure. Therefore, it would have been unethical to conduct the health survey. Transient elastography is an effective non-invasive alternative for evaluating fatty liver and liver fibrosis [37]; therefore, we used it in this study. Second, we used the FAST score to evaluate fibrosis. Although the FAST score can identify patients at risk of progressive NASH, elevated NAFLD activity, and significant fibrosis (score ≥2) in NAFLD patients, its ability to pick up the patients with MAFLD and NAFLD in the general population has not been established. However, a recent cohort study demonstrated the FAST score can be used to stratify disease severity of MAFLD and NAFLD in health examination [21]. In the current study, most of subjects with positive FAST score were diagnosed as having either MAFLD or NAFLD. Additionally, the FAST score alone was used to evaluate patients with MAFLD and NAFLD.

## Conclusions

In a resident health survey, participants with MAFLD had a higher proportion of fatty liver than those with NAFLD. The MAFLD criteria could help identify participants with liver fibrosis at a higher rate. Therefore, the MAFLD criteria could be a useful diagnostic tool for aggressively identifying participants at high risk of fatty liver. Furthermore, obesity and metabolic dysregulation are significantly associated with liver fibrosis. The MAFLD criteria, which include both obesity and metabolic dysregulation, are considered useful in detecting liver fibrosis. In the microbiota analysis, *Blautia* might be one of the causes of MAFLD.

## Supporting information

S1 Dataset (XLSX)
